# Lack of *ROS1* Gene Rearrangement in Glioblastoma Multiforme

**DOI:** 10.1371/journal.pone.0137678

**Published:** 2015-09-14

**Authors:** Sun Min Lim, Junjeong Choi, Jong Hee Chang, Jinyoung Sohn, Kristine Jacobson, Frank Policht, John Schulz, Byoung Chul Cho, Se Hoon Kim

**Affiliations:** 1 Department of Internal Medicine, Division of Medical Oncology, Yonsei University College of Medicine, Seoul, Korea; 2 Department of Pharmacy, College of Pharmacy, Yonsei University, Seoul, Korea; 3 Department of Neurosurgery, Yonsei University College of Medicine, Seoul, Korea; 4 JE-UK Institute for Cancer Research, JEUK Co., Ltd., Gumi-City, Kyungbuk, Korea; 5 Abbott Molecular Diagnostics, Des Plaines, Illinois, United States of America; 6 Department of Pathology, Yonsei University College of Medicine, Seoul, Korea; Oregon State University, UNITED STATES

## Abstract

Glioblastoma multiforme (GBM) is the most aggressive type of brain tumor, and the prognosis remains poor. Rearrangement of *ROS1* gene, which was shown to have an oncogenic potential, was previously discovered in GBM cell lines. In this pilot study, we aimed to identify the incidence of *ROS1* rearrangement in GBM patient tissues to explore novel biomarkers for therapeutic strategy. Formalin-fixed and paraffin-embedded (FFPE) tissue sections from 109 patients with GBM were screened for *ROS1* rearrangement by anti-ROS immunohistochemistry (IHC) and *ROS1* break-apart fluorescent *in situ* hybridization (FISH) assays. O^6^-methylguanine-DNA methyltransferase (*MGMT*) gene promoter methylation and Isocitrate dehydrogenase 1 (*IDH1*) mutation status were also assessed. All samples were interpreted by two experienced pathologists who were blinded to the clinical data. A total of 109 samples were collected and all samples were examined for *ROS1* rearrangement by IHC and FISH assays, and none was found to harbor *ROS1* rearrangement. *MGMT* gene methylation was found in 42 (39.2%) cases, and *IDH1* mutation was found in 6 (5.5%) cases. In this study, *ROS1* rearrangement was not identified in GBM patients, and thus it is difficult to classify *ROS1* rearrangement as a novel molecular subset in GBM patients for now.

## Introduction

Glioblastoma multiforme (GBM) is the most common type of primary brain tumors and the most aggressive subtype of high-grade gliomas. It is classified as grade IV in the World Health Organization classification of tumors of the central nervous system [1, 2]. The current standard treatment strategy for GBM patients consists of surgery followed by concurrent adjuvant radiotherapy in combination with temozolomide. However, still less than 5% of patients survive longer than 5 years after diagnosis. The median overall survival is only 14.6 months with radiotherapy plus temozolomide and 12.1 months with radiotherapy alone [3].

During recent years, comprehensive molecular profiling studies have broadened our knowledge of the underlying genetic and epigenetic aberrations that are associated with initiation and progression of GBM. The incidence of chromosomal rearrangements such as interchromosoal, intrachromosoal and intragenic rearrangements is significantly higher in GBM than in other tumor types [4]. For instance, epidermal growth factor receptor (*EGFR*) was shown to be amplified in 43% of adult GBM, and intragenic deletions in *EGFR* associated with amplification were commonly found in GBM [5, 6]. Besides chromosomal aberrations, frequent mutations of *PTEN* (29%), *TP53* (29%), *EGFR* (20%), *NF1* (9%), *RB1* (8%), phosphatidylinositol-4, 5-bisphospate 3-kinase, catalytic subunit-α (*PIK3CA*; 7%), and *IDH1* (5%) have been reported [7]. However, majority of drugs that target key signaling pathways of GBM have not proved a significant survival benefit in previous GBM patient cohorts [8].

Regarding epigenomic aberrations, the promoter methylation status of the O^6^-methylguanine-DNA methyltransferase (*MGMT*) gene has been suggested as a distinct subset of GBMs. Epigenetic *MGMT* gene silencing by promoter methylation is associated with loss of MGMT expression and diminished DNA repair activity, which leads to increased sensitivity to temozolomide, and thus longer survival [9–11].

*ROS1* is a receptor tyrosine kinase of the insulin receptor family with constitutive kinase activity. *ROS1* was found to be expressed in most glioblastoma cell lines, and characterization of ROS1 cDNA revealed a structural class of transmembrane protein kinase [12–14]. Rearrangement of *ROS1* gene, involving *ROS1* carboxy-terminal kinase fused to the amino-terminal portion of a protein called FIG (Fused in Glioblastoma) was also found in glioblastoma cell line [15]. When 10 different cell lines from all grades of astrocytomas were screened for this fusion transcript, *FIG-ROS1* was found in two GBM cell lines (U118MG and U138MG).Interestingly, this *FIG-ROS1* fusion transcript retained the active kinase domain with oncogenic potential. Recently, Stransky et al. reported the identification of *CEP85L-ROS1* in a glioblastoma patient sample [16].

*ROS1* rearrangement was also found in other solid tumors such as non-small-cell lung cancer (NSCLC) and cholangiocarcinoma [17–20]. To date, nine different *ROS1* fusion partners have been identified in NSCLC, all of which are potentially targetable due to the same cytoplasmic portion of the ROS1 tyrosine kinase domain [21]. Due to the biologic similarity of *ROS1* and *ALK*, several *ALK* inhibitors have been shown to inhibit *ROS1* [22]. Preliminary data from a phase 1 trial of crizotinib in the *ROS1*-positive NSCLC expansion cohort demonstrated an overall response rate of 61% [23]. Therefore, identification of *ROS1* rearrangement in GBM could offer a new therapeutic option to tackle this fatal disease.

In this study, we aimed to identify the incidence of *ROS1* rearrangement and evaluate clinicopathological features associated with *ROS1* rearrangement in GBM patients.

## Methods

### Patient characteristics

Patients with histologically proven GBM (World Health Organization grade 4) with newly diagnosed GBM were identified consecutively between January 2001 and December 2013. Of these, 109 patients who had available tissue samples for biomarker analyses were selected for this study. All patients provided written informed consent. Study protocol and informed consent forms were approved by the ethics committee and the institutional review board of Severance Hospital.

### *ROS1* Fluorescence *in situ* Hybridization

To identify *ROS1* rearrangement, fluorescent *in situ* hybridization (FISH) assays were carried out on formalin-fixed and paraffin-embedded (FFPE) tumors by using a break-apart probe to *ROS1* (Break-Apart Rearrangement Probe; Abbott Molecular) according to manufacturer’s instructions. At least 100 nuclei per case were evaluated. FISH positivity for *ROS1* rearrangement was defined as > 15% of tumor cells with a split signal. FISH studies were interpreted by two experienced evaluators (SHK & JC) who were blinded to the clinical data.

### ROS1 immunohistochemistry

For ROS1 immunohistochemistry (IHC) analysis, FFPE tissues sectioned at a thickness of 4uM and stained using Ventana automated immunostainer BenchMark XT. The slides were dried at 60°C for 1h and deparaffinized using EZ Prep at 75°C for 4 min. Cell conditioning was carried out using CC1 solution at 100°C for 64 min. ROS1 antibody (rabbit monoclonal, clone D4D6, Cell Signaling Technology) was diluted to 1:50, treated, and incubated at 37°C for 32 mins. Signals were detected using OptiView DAB IHC Detection Kit (Ventana Medical Systems). Counterstaining was carried out with Hematoxylin for 4 min at room temperature. Immunostained slides were scored with intensities of 0, 1+, 2+, and 3+ as follows: intensity 0 was defined as no detectable staining. Intensity 1+was defined as reactivity only detectableat high magnification (x 20–40 objective). More intense reactivity was divided into moderate(2+) and strong (3+) based on the ease of detectionat low magnification (x 4 objective).For interpretation of ROS1 expression, 3+ perinuclear staining was considered positive. IHC studies were interpreted by two experienced evaluators (SHK & JC) who were blinded to the clinical data.

### *MGMT* gene promoter methylation assay

*MGMT* gene promoter methylation was assessed in patients with available tissue. Genomic DNA was extracted from 107 paraffin-embedded samples and the DNA methylation status of CpG islands at the *MGMT* promoter was assessed by methylation-specific polymerase chain reaction as previously described [11]. Unmethylated control DNA and methylated control DNA with bisulfite treatment (Qiagen, Germany) were used as negative and positive controls, respectively. Polymerase chain reaction products were separated on 8% polyacrylamide gels, stained with ethidium bromide, and examined under ultraviolet illumination by investigators blinded to clinical information.

### Isocitrate dehydrogenase 1 (*IDH1*) sequencing analysis

IDH1 assay was performed according to the method described by previously [24]. DNA was isolated from each FFPE tumor tissue using a QIAamp DNA FFPE tissue kit (Qiagen, Valencia, CA, USA) according to the manufacturer’s instructions. The quantity of isolated genomic DNA was evaluated using a NanoDrop 1000 spectrophotometer (Thermo Scientific, Wilmington, DE, USA). The detection of IDH1 mutation was performed by polymerase chain reaction (PCR) using forward and reverse primers that were designed to amplify exon 4 (codon R132) of the IDH1 gene. IDH1 forward primer (5’-ACC AAA TGG CAC CAT ACG A-3’) and reverse primer (5’-GCA AAA TCA CAT TAT TGC CAA C-3’) generated a 130-bp PCR product. PCR amplification was performed using an AmpliTaq Gold PCR Master Mix (Applied Biosystems, Foster City, CA, USA). The reaction mixture was subjected to an initial denaturation at 95 C for 10 min, followed by 35 cycles of amplification consisting of denaturation at 95 for 30s, annealing at 55 C for 30 s, and extension at 72 C for 60 s. After purification and sequencing amplification, the sequencing products were analyzed by a 3730XL DNA sequencer (Applied Biosystems).

### Cell line

For positive control of *ROS1* FISH assay, U118MG cell line (ATCC, Manassas, VA) maintained in Dulbecco’s modified Eagle’s medium supplemented with 10% FBS and the 1% antibiotics streptomycin at 37°C in a 5% CO2 environment, was used.

### Statistical analysis

Progression-free survival (PFS) and overall survival (OS) were measured from the time of surgery to disease progression or death, or date of last follow-up visit, and were analyzed using the Kaplan-Meier method. Log-rank test was used to compare *MGMT* promoter methylation status with survival. Cox proportional hazards model was used to perform univariate and multivariate analyses. *P* values of ≤ 0.05 were considered statistically significant.

## Results

### Patient characteristics

**Fig 1:**
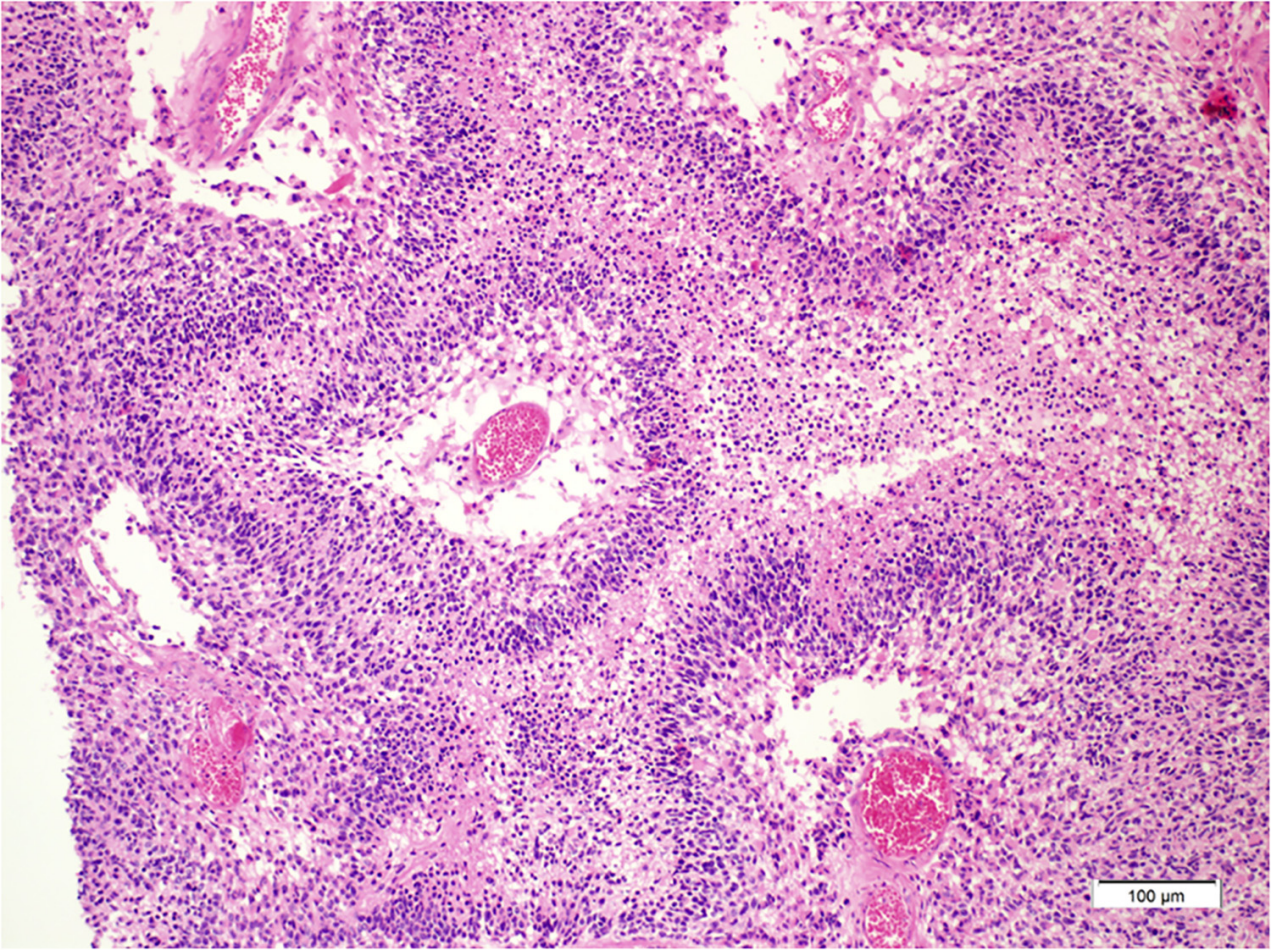
Classical histologic findings of glioblastoma showing prominent pseudopalisading necrosis are seen (hematoxylin and eosin x 200).

**Fig 2:**
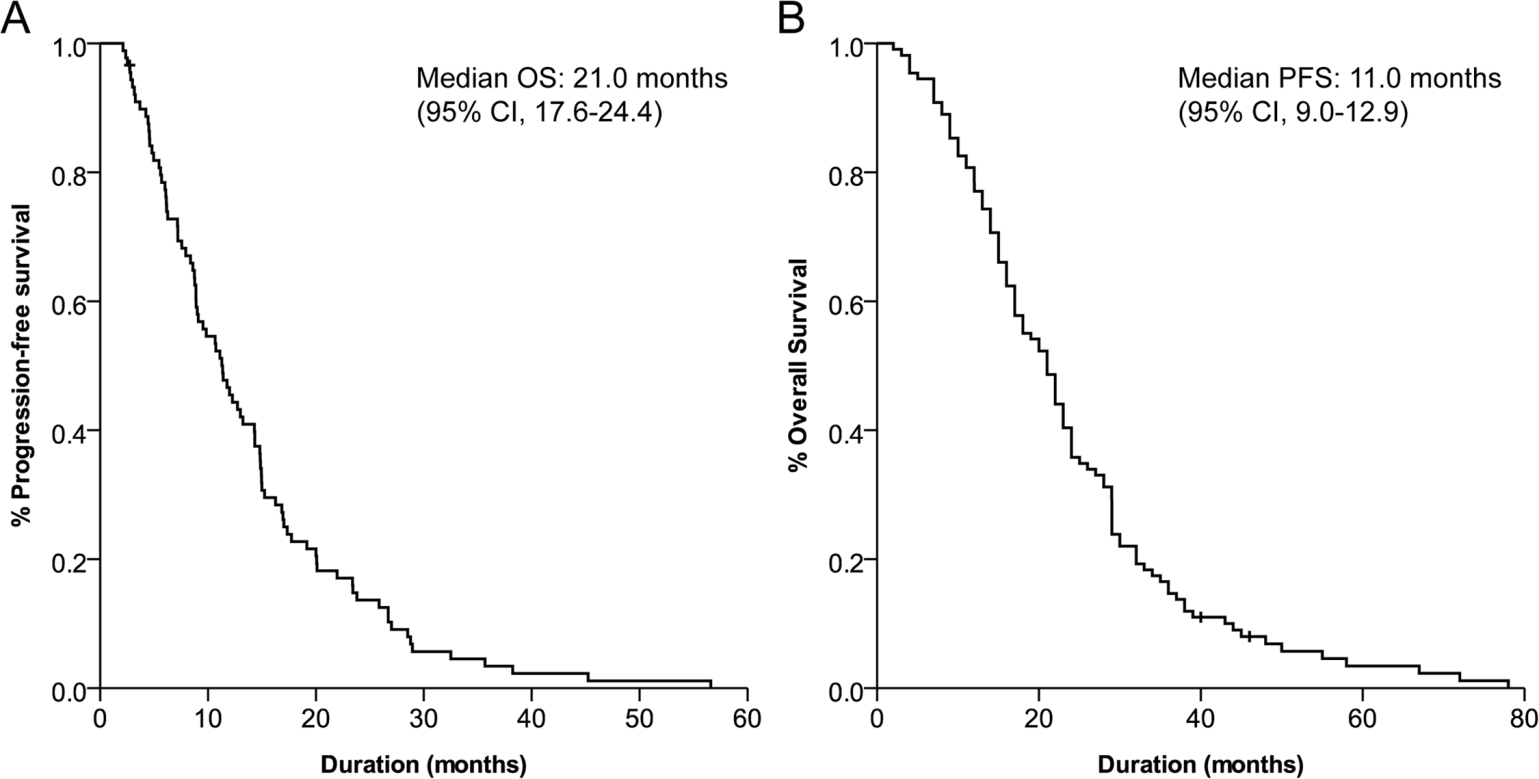
Kaplan-Meier curve of (A) overall survival and (B) progression-free survival of all patients.

A total of 109 patient samples with histologically proven GBM were available for analysis (Table 1, Fig 1). The median age of all patients was 56 years (range, 20–84 years), and there were 61 males (56%) and 48 females (48%). Total surgical resection was performed in 82 patients (75%), partial resection in 20 patients (18%) and biopsy was performed in 7 patients (7%). With the median follow-up period of 24 months, the median OS was 21.0 months (95% CI, 17.6–24.4) and the median PFS was 11.0 months (95% CI, 9.0–12.9) (Fig 2A and 2B).

**Table 1:** Clinicopathological characteristics of patients (n = 109).

Characteristics	No (%)
Age, years	
Median	56
Range	20–84
Sex	
Male	61 (56)
Female	48 (44)
Surgery	
Biopsy	7 (6.5)
Partial resection	20 (18.3)
Total resection	82 (75.2)
*ROS1* rearrangement	
Yes	0 (0)
No	109 (100)
*MGMT* promoter status	
Methylated	42 (38.5)
Unmethylated	65 (59.6)
Unknown (invalid, indeterminate)	2 (1.9)
*IDH1* mutation	
Mutated (R132H)	6 (5.5)
Wild type	103 (94.5)
Overall survival (months)	21
95% CI	17.6–24.4
Progression-free survival (months)	11
95% CI	9.0–12.9

Abbreviations: *MGMT*, O^6^-methylguanine-DNA methyltransferase; *IDH1*, Isocitrate dehydrogenase 1

### Analysis of *ROS1* rearrangement

**Fig 3:**
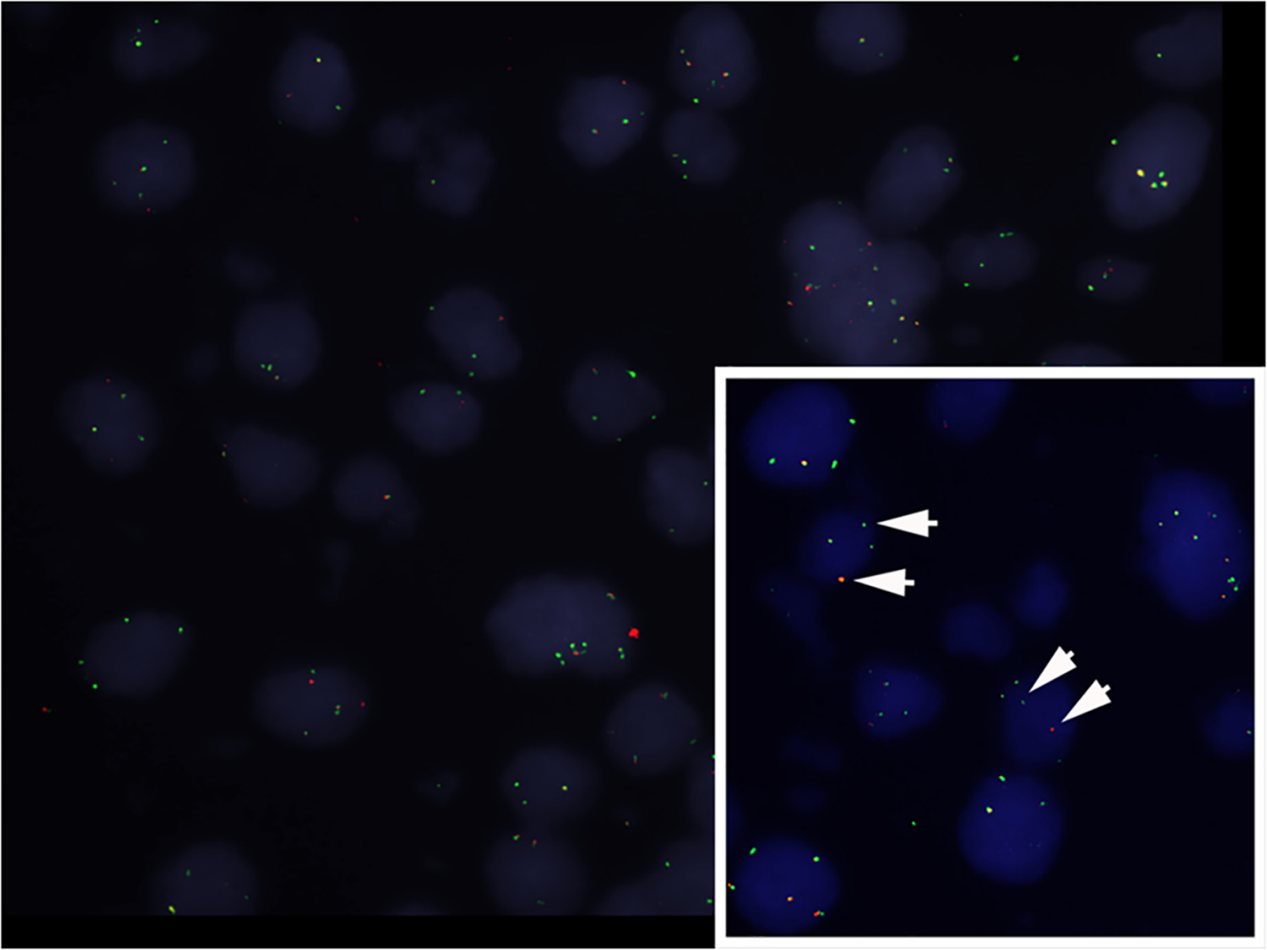
*ROS1* break-apart fluorescent *in situ* hybridization shown in U118MG cells.

**Fig 4:**
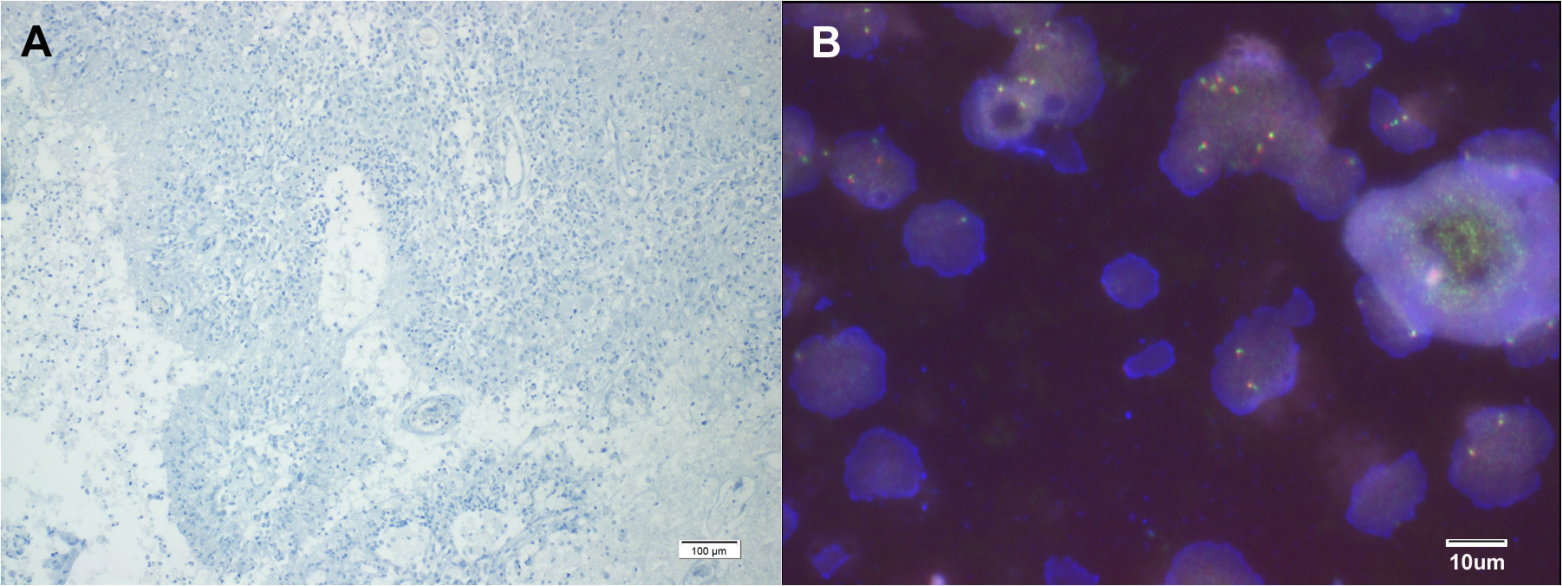
(A) Immunohistochemical staining of ROS1 showing negative immunoreactivity (B) *ROS1* break-apart fluorescent *in situ* hybridization showing negativity for *ROS1* rearrangement.

FISH analysis of U118MG showed *ROS1* rearrangement (Fig 3). We performed IHC in 109 GBM patient samples and there was no positive staining for *ROS1* (Fig 4A). Next, we tested with *ROS1* FISH break-apart probes, but none met the criteria to be considered FISH-positive. No sample had separation of 5' (green) and 3' (red) signals, and no sample had isolated 3' (red) signals detected (Fig 4B).

### Analysis of *MGMT* methylation and *IDH1* mutation

Among 107 patients whose *MGMT* methylation status was available, 42 (38.5%) patients had methylated *MGMT* promoter and 65 (59.6%) patients had unmethylated *MGMT* promoter. The methylation status could not be determined in the remaining 2 (1.9%) patients (S1 Fig). *IDH1* mutation was found in 6 (5.5%) patients, all of whom had point mutations affecting codon 132 of the *IDH1*, located on chromosome locus 2q33. This mutation resulted in arginine to histidine substitution (R132H mutation) in all four samples (S2 Fig).

### Prognostic factors of survival

We analyzed univariate and multivariate analysis to identify prognostic factors of OS. Age, sex, extent of resection and *MGMT* gene promoter methylation status were included in analysis. Univariate analysis revealed that age, extent of resection, and *MGMT* gene promoter methylation status were significant prognostic factors. Multivariate analysis revealed that age and *MGMT* gene promoter methylation status were independent prognostic factors for OS (Table 2).

**Table 2:** Univariate and multivariate analyses of prognostic factor of overall survival.

		Univariate analysis			Multivariate analysis	
		Overall survival			Overall survival	
Variable	No. of patients	Median, month (95% CI)	HR (95% CI)	*P*	HR (95% CI)	*P*
Age (y)						
≤50	42	23 (17–28)		**0.033**		**0.005**
>50	67	17 (14–21)	1.54 (1.04–2.31)		1.71 (1.14–2.58)	
Sex						
M	61	19 (15–22)		0.988		-
F	48	17 (11–22)	0.99 (0.68–1.47)		-	
Extent of resection						
Total resection	82	21 (16–25)		**0.04**		**0.022**
Partial + biopsy	27	15 (12–17)	1.45 (1.04–2.32)		1.76 (1.09–2.86)	
*MGMT* gene						
Methylated	42	24 (19–28)		**0.031**		**0.009**
Unmethylated	65	17 (15–18)	1.55 (1.04–2.32)		1.72 (1.14–2.59)	
Unknown	2	5 (4–7)	-		-	

## Discussion

In this study, we screened *ROS1* rearrangement in GBM patients by both FISH and IHC for the first time, and reported that *ROS1* rearrangement was not discovered in this GBM cohort. To date, *ROS1* rearrangement has been only identified in GBM cell lines and it is unclear if other fusion variants exist in clinical samples.

Treatment of patients with GBM evolved slowly in the last decades. Although genetic and epigenetic alterations have been found in GBM, drugs that specifically target signaling pathways such as receptor tyrosine kinase have not proved a significant benefit in survival in unselected GBM patient cohorts [25]. Bevacizumab, an angiogenesis inhibitor, showed in two recent randomized phase III trials to bring about 3 to 4month prolongation of progression-free survival, without significant effect on overall survival [26, 27]. Therefore, efforts to find genetic alterations that drive gliomagenesis and identify molecularly defined patient subgroups for targeted therapies are imperative.

The discovery and characterization of *ROS1* rearrangement in solid tumors have raised significant clinical interest because small molecule inhibitors may be effective to these tumors. Currently, 9 fusion partners to *ROS1* have been identified (*FIG*, *CCDC6*, *CD74*, *EZR*, *KDELR2*, *LRIG3*, *SLC34A2*, *SDC4*, *TPM3*) all of which retain the ROS1 cytoplasmic kinase domain. The oncogenic *ROS1* gene fusion in lung adenocarcinomas, which is identified in up to 3.4% of patients, has expanded the list of themolecular subsets of lung cancers [21–23, 28–30]. Since *ALK* and *ROS1* share an approximately 49% amino acid sequence in the kinase domain, ALK inhibitors have been proved to be effective in inhibiting *ROS1* activity [22]. Currently, there are ongoing clinical trials of drugs targeting *ROS1* for non-small cell lung cancer patients with *ROS1* rearrangement.

For cholangiocarcinoma (CCA), only *FIG-ROS1* fusion transcript has been identified so far. Gu *et al*. found out the presence of *ROS1* rearrangement in 8.7% of CCA patients and demonstrated inhibition of growth by ALK inhibitor in *ROS1* rearranged CCA cells. These results suggest that *ROS1* rearrangement in CCA is a promising druggable target with considerable incidence [20]. *ROS1* rearrangement was also found in gastric cancer patients where IHC analysis revealed 23 (4.6%) positive cases among which 3 (0.6%) were FISH positive [31].

In our study, we used both FISH and IHC analyses for screening because RT-PCR method can only detect known fusion variants. For screening methods, FISH and reverse transcriptase-polymerase chain reaction (RT-PCR) have been used more commonly, although they are time consuming, costly, and not suitable for rapid screening. Immunohistochemical analyses using an anti-ROS1 rabbit monoclonal antibody (D4D6) have recently shown to accurately identify *ROS1*-rearranged cancers showing 100% (8/8) sensitivity and 100% (138/138) specificity when compared with break-apart FISH [28]. To date, only *FIG-ROS1* has been identified in GBM cell lines and it is unclear whether *ROS1* fusion variants with oncogenic activity exist in clinical samples.

The limitation of this study arises from a relatively small sample size. Reflecting upon the incidences of *ROS1* rearrangements found in other tumor types, it is a rare phenomenon that requires large-scaled screening efforts. Moreover, there may be a possibility of false-negative IHC test results due to a low level of the expressed *ROS1* fusion transcripts.

Although *ROS1* rearrangement was not identified in our study cohort, it is notable that the initial discovery of *ROS1* rearrangement in NSCLC was based on the identification in 1 cell line [17]. It is uncertain that *ROS1* rearrangement may represent a potentialnew therapeutic opportunity for now, but biomarker discovery efforts should be continued to develop molecular tumor classification and to improve outcome and management of patients with GBM.

## Supporting Information

S1 Fig *MGMT* methylation analysis showing methylation in case 1, and unmethylation in case 2 and case 3 patients. (TIF)

S2 Fig Mutation found at codon 131 (GCT) of isocitrate dehydrogenase enzyme isoforms (*IDH*)1 resulting in Arg132His (c.395G>A) change. (TIF)
