# Supplementary figures and images for: Lack of ROS1 Gene Rearrangement in Glioblastoma Multiforme
Source: PLoS One. 2015 Sep 14;10(9):e0137678. doi: 10.1371/journal.pone.0137678 (PMC4569301; doi:10.1371/journal.pone.0137678)

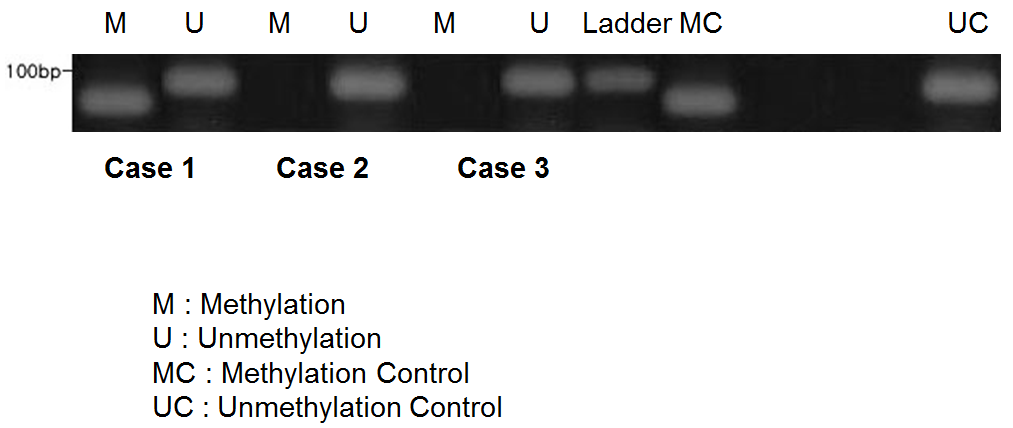

Supplement: S1 Fig — (TIF) [file pone.0137678.s001.tif]

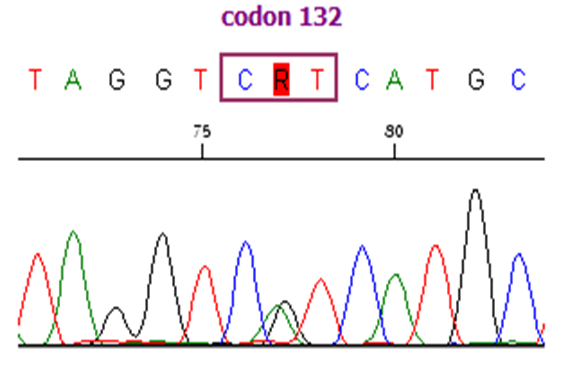

Supplement: S2 Fig — (TIF) [file pone.0137678.s002.tif]
